# Retroflexion of the Gravid Uterus

**Published:** 1900-12-22

**Authors:** 


### RETROFLEXION OF THE GRAVID UTERUS.

Dr. W. J. Sinclair speaking at the Obstetrical Society on the subject of the Diagnosis and Treatment of the Gravid Uterus, pointed out in the first place that the striking constant feature of this condition was irritability of the bladder with more or less retention of urine. Then in regard to treatment he advocated a method which was extremely simple, and which had in his hands proved extremely effectual. It consisted essentially in the introduction of a watch-spring pessary, care having previously been taken to empty the bowel and bladder. After the introduction of the pessary, if the patient were made to rest on her side, lying over with her face downward as far as she could with comfort, it would be found, he said, that the action of the pessary alone would restore the uterus to its normal position. Dr. Sinclair had tried this plan in fifteen cases which had been consecutive, and in all of which it had been successful.

